# Cerebral Neovascularization and Remodeling Patterns in Two Different Models of Type 2 Diabetes

**DOI:** 10.1371/journal.pone.0056264

**Published:** 2013-02-18

**Authors:** Roshini Prakash, Maribeth Johnson, Susan C. Fagan, Adviye Ergul

**Affiliations:** 1 Charlie Norwood Veterans Administration Medical Center, University of Georgia College of Pharmacy, Augusta, Georgia, United States of America; 2 Program in Clinical and Experimental Therapeutics, University of Georgia College of Pharmacy, Augusta, Georgia, United States of America; 3 Department of Biostatistics, Georgia Health Sciences University, Augusta, Georgia, United States of America; 4 Department of Physiology, Georgia Health Sciences University, Augusta, Georgia, United States of America; University of South Florida, United States of America

## Abstract

We previously reported intense pial cerebral collateralization and arteriogenesis in a mild and lean model of type 2 diabetes (T2D), Goto-Kakizaki (GK) rats. Increased cerebral neovascularization differed regionally and was associated with poor vessel wall maturity. Building upon these findings, the goals of this study were to determine whether a) glycemic control prevents this erratic cerebral neovascularization in the GK model, and b) this pathological neovascularization pattern occurs in Lepr^db/db^ model, which is the most commonly used model of T2D for studies involving cerebral complications of diabetes. Vascular volume, surface area and structural parameters including microvessel/macrovessel ratio, non-FITC (fluorescein) perfusing vessel abundance, vessel tortuosity, and branch density were measured by 3D reconstruction of FITC stained vasculature in GK rats or Lepr^db/db^ mice. GK rats exhibited an increase in all of these parameters, which were prevented by glycemic control with metformin. In Lepr^db/db^ mice, microvascular density was increased but there was no change in nonFITC-perfusing vessels. Increased PA branch density was associated with reduced branch diameter. These results suggest that T2D leads to cerebral neovascularization and remodeling but some structural characteristics of newly formed vessels differ between these models of T2D. The prevention of dysfunctional cerebral neovascularization by early glucose control suggests that hyperglycemia is a mediator of this response.

## Introduction

Diabetes-mediated microvascular disease of the brain is increasingly recognized as a risk factor for neurodegenerative diseases like vascular cognitive impairment and stroke [1]. Changes in cerebrovascular structure and function can lead to altered blood brain barrier (BBB) permeability and cerebral blood flow not only contributing to the development of the disease but also impairing the recovery after an ischemic event like stroke [2]. Earlier we showed greater pial cerebral arteriogenesis characterized by increased collateralization and tortuosity in the Goto-Kakizaki (GK) model of diabetes [3], [4]. This model also develops new cerebral blood vessels that have greater permeability and reduced wall maturity thus making them liable to bleeding during an ischemic insult [4], [5]. It is known that vascular morphogenesis in a healthy animal is dependent upon physiologic, metabolic and local factors [6]. The presence of a disease state and its severity affects these factors in relation to the altered functional needs of a particular tissue [7]. Therefore, understanding of the cerebrovascular architecture that is important for the delivery of oxygen and nutrients in different experimental models of diabetes is critical to identify and develop novel therapeutic targets for prevention and treatment of cerebral microvascular complications in diabetes. Building upon our earlier work, the current study sought to answer the following questions: 1) Is glycemic control effective in the prevention of dysfunctional cerebral angiogenesis in the GK model of diabetes?, and 2) Are the pathological changes observed in the GK model present in Lepr^db/db^ model of T2D, the most commonly used model for studies involving cerebral complications of diabetes including stroke and cognitive impairment.

## Methods

### Animal Preparation and Glycemic Control

All procedures on animals for the study were carried out in accordance with National Institute of Health guidelines for the care and use of animals in research and under protocols approved by the Georgia Health Sciences University. Glycemic control in the GK rats was achieved using metformin (150–300 mg/kg body weight, dose escalated with age to a maximum of 300 mg/kg) in artificially sweetened drinking water to mask the metallic taste of the drug. Euglycemia was targeted in GK rats starting at 6 weeks of age at the onset of diabetes until 11 weeks when established vascular disease is seen in the untreated parallel GK group. The control group was not treated with metformin due to ethical reasons as they begin to lose weight and deteriorate upon treatment. Metabolic parameters are given in Table 1.

**Table 1:** Metabolic parameters of both the diabetic models.

Groups/Parameters	Body Weight (g)	Blood Glucose (mg/dL)
**Control (Wistar), n = 8**	301±2	98±3
**Diabetes (GK), n = 8**	285±5	183±8*
**Diabetes+Metformin, n = 6**	291±4	96±3
**Control (c57Bl), n = 5**	29±1	97±3
**Diabetes (Lepr ^db/db^), n = 5**	61±1*	306±31*

* p<0.001 vs control.

### Imaging of Cerebral Vasculature

Vascularization patterns and density were measured using the space-filling FITC-Fluorescein IsoThioCyanate-dextran method as we recently described. Brains were processed in 4% paraformaldehyde (24 h) and 30% sucrose in phosphate-buffered saline (PBS), sectioned into 100 µm slices and mounted on slides. Z-stacked confocal images of the regions proximate to the middle cerebral artery (MCA) and its branches that supply the frontal motor cortex, bregma 1 to −1 were acquired using Zeiss LSM 510 upright confocal microscope. Cortical parenchymal vessels that dive in from the surface vessels and its immediate first order branches were imaged at 10X in this region. A mean of 3 values from this region was recorded as an observation. Each measurement from one animal was comprised of an average of 9 images from either the cortical or striatal region. Retinal flat mount, gastrocnemius and soleus muscles were prepared similarly in the same animals to assess neovascularization in different vascular beds. Axial distance of 1 µm spaced images was obtained using 25X objective to assess 3 dimensional parameters.

### Indices of Neovascularization

*Vascular volume* refers to the ratio of the volume of the FITC stained vasculature to the total volume (reference volume) of the section on a Z-stack as this represents the unit volume of blood being supplied to the brain tissue in the region of interest [5], [8]. Absolute *surface area* represents the area available for the diffusion/exchange of vascular nutrients to the surrounding brain tissue [8], [9]. Penetrating arterioles (PA) were defined as the macrovessels that immediately penetrate perpendicularly into the cerebral cortex. The immediate first order branches from the PA were termed as the PA1. PA and PA1 were selected on each image using Volocity Improvision and represented the macrovasculature. Their respective volumes and surface area measurements were derived. Microvascular measurements were obtained by subtracting the macrovascular measures (PA and PA1) from the total vascular parameters (Supplementary Figure 1).

**Figure 1:**
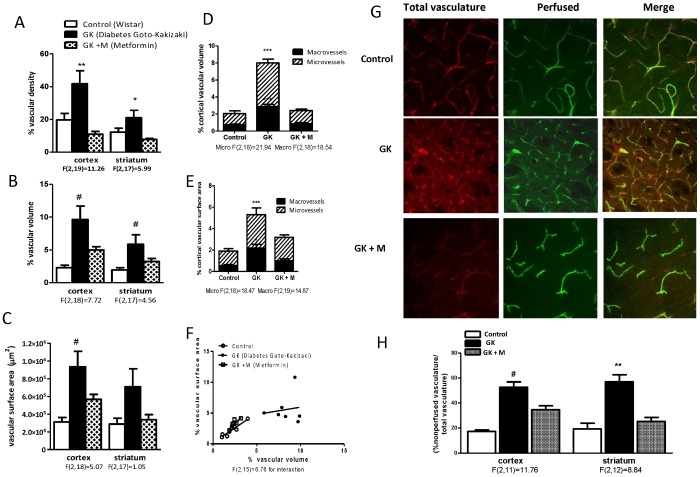
Glycemic control prevents neovascularization in the GK model of diabetes. (A) Vascular density is significantly increased in both cortex and striatum of GK rats and this was prevented by metformin treatment started at the onset of diabetes. (B) Vascular volume and (C) vascular surface area are also increased in diabetes. (D) Both micro and macrovascular volume as well as surface area (E) are increased in GK rats as compared to control. (F) Diabetic vascular correlations are extremely disproportionate and the slopes of the two lines are significantly different. (G, H) Immature cerebral microvessels are more abundant in diabetes. Representative images of cerebral striatal vasculature showing perfused (green-FITC dextran) and non-perfused vessels (Red-Isolectin). GK rats exhibit increased immature vasculature and glycemic control with metformin reduced the immature vasculature. F values are indicated under each ANOVA analysis group. *p<0.05 vs treatment, **p<0.01 vs control or treatment, # p<0.05 vs control, ***p<0.001 vs control or treatment. Mean ± SEM, n = 6−8.

To differentiate vessels not perfused with FITC, brain sections were co-stained with biotinylated isolectin B4 (Vector Laboratories Inc. Burlingame, CA) that binds to basement membrane and marks all endothelial cells [5]. Isolectin stained the entire vasculature both perfused and non-perfused. A difference between the isolectin-stained entire vasculature and the FITC perfused vasculature represented the non-perfused vessels.

Vessel structure and morphometry was assessed using Fiji software and axially projected into an 8-bit image [10]. Centerlines reduced to 1-pixel size were extracted to obtain binary skeletonized images, which were then assessed to determine the tortuosity, diameter of the PA and PA1, and the number of branch points associated with the penetrating arterioles. For vessel tortuosity, the centerline line extracted images were analyzed by longest-shortest distance method without pruning the ends to measure the actual length of the vessels [11], [12]. A ratio of this value over the euclidean distance provided the *tortuosity* or skewness of the vessel. The values obtained from this analysis were sorted in a descending fashion. *Diameter* measurements were drafted manually as the parallel distance between internal walls of the vessels after outlining the lumen using Fiji software. An average of 3–4 values was recorded as mean diameter of a given vessel to assess remodeling of the lumen. *Branch density* refers to the number of branch points found over unit length of a vessel [13], [14]. (Methodology shown on Supplementary Figure S1).

### Astrocytic Structure

The FITC stained sections were co-stained with glial fibrillary acidic protein (GFAP) (Millipore). Surface area of the GFAP stained astrocytes were determined. A ratio of the surface area over the total number of astrocytes in the image was evaluated as the astrocytic surface density.

### Statistical Analysis

Data are expressed as mean±SE. Data were evaluated for normality and appropriate transformations were used when necessary. One-way ANOVAs on the ranks of the data were used to compare control (Wistar), Diabetes (GK), and GK+Metformin groups of rats for all variables. A Tukey’s test was used to adjust for multiple comparisons for significant group effects. t-tests on the ranks of the data were used to compare control (C57BL) and diabetic (Lepr^db/db^) mice for all variables. A test for the homogeneity of slopes among the groups of rats or mice for the relationship between percent vascular surface area and percent vascular volume was performed using ANCOVA on the ranked data. Statistical significance was determined at alpha = 0.05 and due to the small sample sizes for some of the variables a statistical trend was determined at alpha = 0.10. F and t values for ANOVA and t-test analyses, respectively, are given in Figure legends. SAS® version 9.3 was used for all analyses (SAS Institute, Inc., Cary, NC).

## Results

### Glycemic Status in the Two Models of Diabetes

Blood glucose was higher in both models of diabetes as compared to their respective controls. Body weight was greater in the Lepr^db/db^ mice than in wild-ype mice (Table 1).

### Cerebral Microvascular and Macrovascular Density in Diabetes

Vascular measurements were made in the cortical and striatal regions that are susceptible to vascular injury when these animals are subjected to focal ischemic brain injury as we reported before. The GK model displayed increased total vascular density, volume and surface area in both cortex and striatum (Figure 1A–C). When the relationship between vascular volume and surface area was analyzed, there was a linear correlation between the surface area and volume in control rats but there was a disproportional increase in these parameters in the GK group, suggesting a contribution from both macro and microvasculature. These results also suggested that in GK rats there were some vessels that are getting larger (more volume) without a parallel increase in area indicative of larger vessel remodeling. We then analyzed the relative density of micro and microvasculature in these sections. As shown in Figure 1D and E, GK rats displayed an increase in both micro and macrovessel volume and surface area. Glucose control with metformin initiated right after onset of diabetes lowered blood glucose levels to control levels in GK rats (Table 1) and completely prevented the increase in vascular density (Figure 1A). Glucose control also corrected the relationship between vascular volume and surface area (Figure 1F).

Since FITC-dextran is a space-filling model, it is assumed that vessels visualized are perfused with FITC. In order to differentiate vessels that are not perfused with FITC, the same sections were labeled with isolectin. The nonperfused/perfused vessel ratio that we reported to be greater in GK rats was normalized with glycemic control (Fig 1G and H).

**Figure 2:**
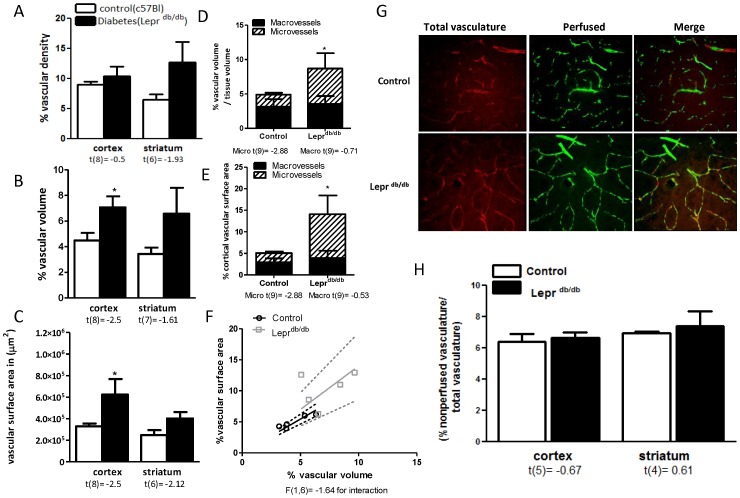
Evidence for neovascularization in the Lepr^db/db^ model of diabetes. (A) Not vascular density but (B) vascular volume and (C) vascular surface area are significantly increased in the cortex of Lepr^db/db^ mice. (D) Cortical microvascular volume and surface area are also increased. (F) Linear regression graph depicts the correlation between the vascular volume and surface area of the vasculature (G, H) There was no difference in the perfused/nonperfused vessel ratio in the Lepr^db/db^ mice in cortex. t values are indicated under each analysis group. *p<0.05 vs control, Mean ± SEM, n = 5.

There was no difference in vascular density in Lepr^db/db^ mice as compared to control mice (Figure 2A). There was, however, an increase in 3-D indices like vascular volume and surface area (Figure 2B and C). Lepr^db/db^ mice showed an increase in microvascular volume and area (Figure 2D and F). The nonperfused/perfused vessel ratio was not different between control and diabetic animals in the Lepr^db/db^ model.

### Cerebral Vascular Tortuosity and Branching in Diabetes

**Figure 3:**
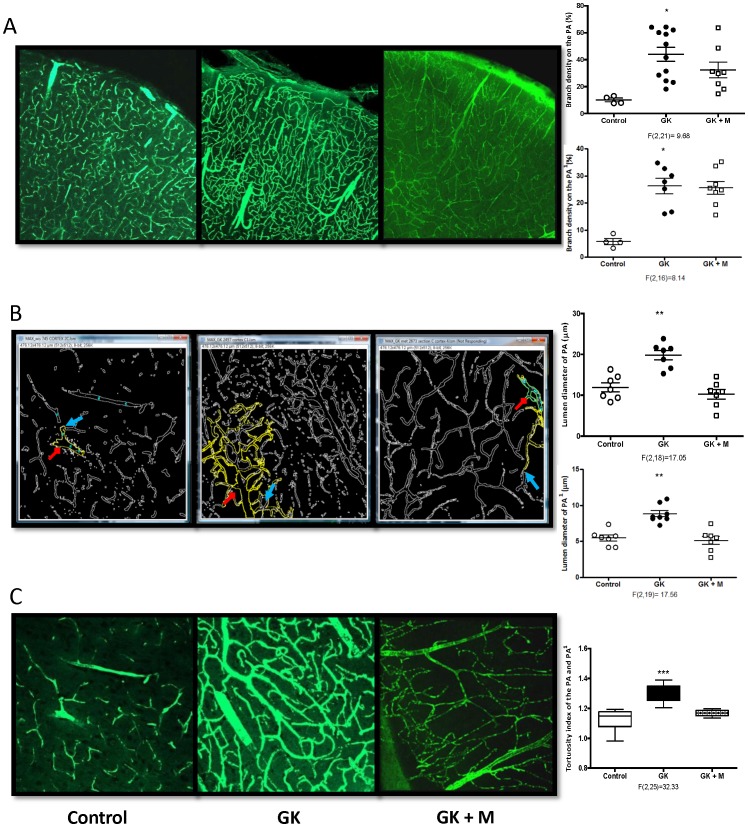
Diabetes increases branch density, lumen diameter and tortuosity of PAs and its subsequent branches in the GK model of T2D. Representative images showing (A) vascular branching on PAs and surface cortical vessels taken under 10X, (B) inner vessel walls outlined using the Fiji software (Red arrows represent the Penetrating arterioles (PA) and the blue arrows depict the immediate branched from the PA (PA^1^), outlined yellow), and (C) tortuous cortical vessels imaged under 25X objective. GK rats exhibit profound increase in branch density, diameter and tortuosity of PA and PA^1^. While there was no significant change in branching of PAs with glycemic control, lumen diameter and tortuosity was reduced. F values are indicated under each ANOVA analysis group. *p<0.01 vs control, **p<0.001 vs control or treatment, ***p<0.0001 vs control or treatment. Mean ± SEM, n = 6−8.

**Figure 4:**
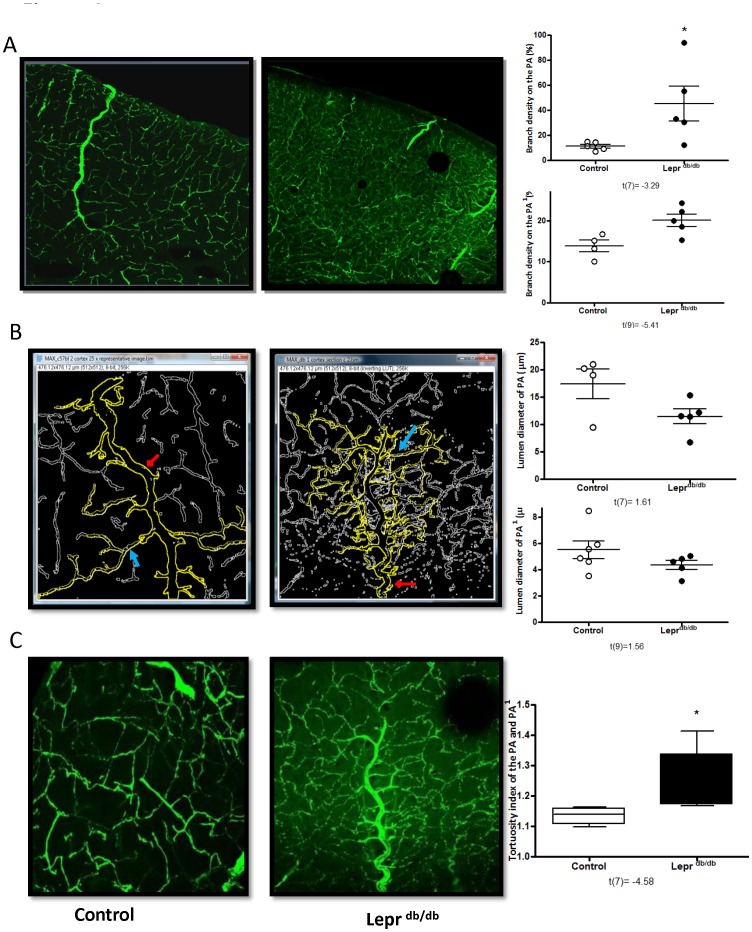
Diabetes increases vascular remodeling. Panel (A) showing increased branch density of PA and PA^1^. (B) There was a trend for decreased lumen diameter. (C) Tortuosity of PAs and its subsequent branches are increased in the db/db model of T2D. t values are indicated under each analysis group. *p<0.05 vs control. Mean ± SEM, n = 5.

GK rats exhibited significantly enhanced branch density of the PAs (Figure 3A). The diameter as well as the tortuosity of these vessels were increased (Figure 3B). Euglycemia achieved by treating with metformin prevented the increases in lumen diameter and tortuosity but did not impact branch density in GK rats (Figure 3A–C). Branch density and tortuosity were enhanced in the db/db model but there was trend for decreased lumen diameter of the penetrating arterioles (Figure 4A–C).

### Peripheral Neovascularization in Diabetes

**Figure 5:**
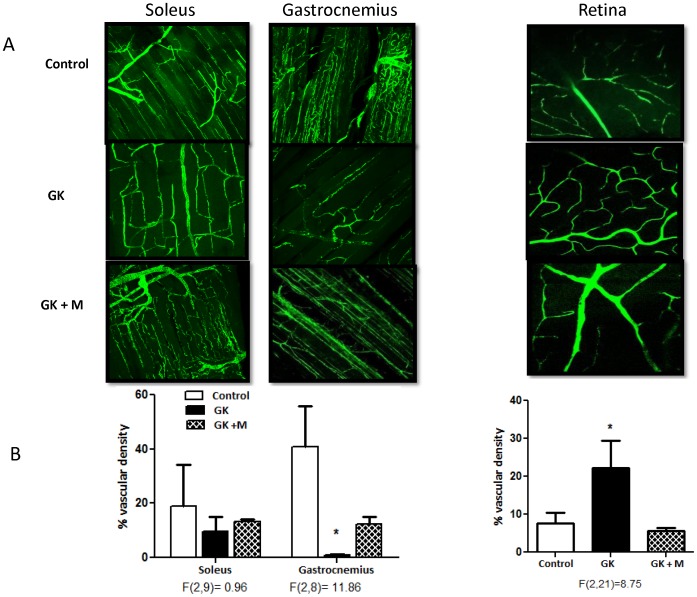
Glycemic control prevents impaired neovascularization in the skeletal muscle and retina in the GK model of diabetes. Representative images of blood vessels in gastrocnemius and soleus muscles as well as in the retina are given on top (A) and cumulative bar graphs are shown at the bottom (B). Glycemic control with metformin restores the peripheral blood vessels and prevents increased retinal vascularization. F values are indicated under each ANOVA analysis group. *p<0.05 vs control or treatment. Mean ± SEM, n = 5−7.

**Figure 6:**
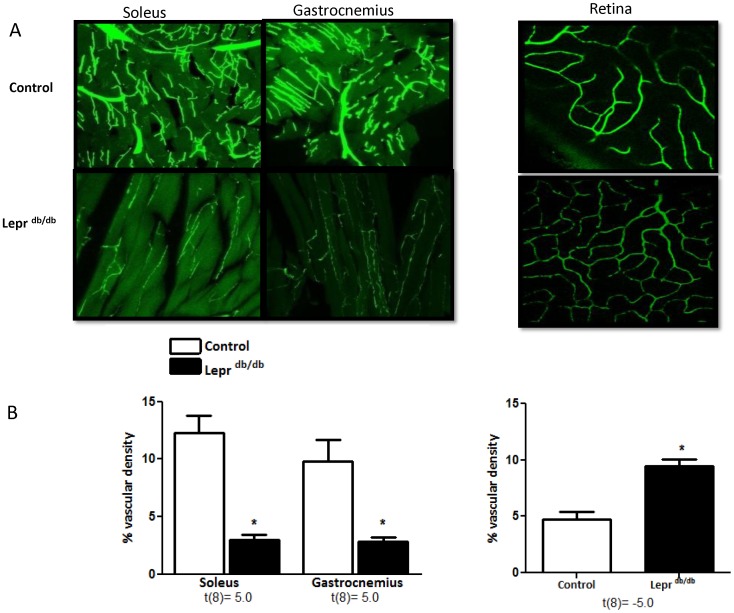
Peripheral vascularization is severely impaired in Lepr^db/db^ mice. Representative images of blood vessels in gastrocnemius and soleus muscles as well as in the retina are given on top (A) and cumulative bar graphs are shown at the bottom (B). t values are indicated under each analysis group. *p<0.05 vs control. Mean ± SEM, n = 5.

The decreased vascular density in the gastrocnemius muscle and the increased retinal vascular density in GK rats were prevented by metformin treatment (Figure 5). Similar changes were noted in the Lepr^db/db^ mice. There was a profound decrease in both soleus and gastrocnemius muscle vascular density and an increase in the retinal vascularization (Figure 6).

### Reactive Astrocytes in Diabetes

**Figure 7:**
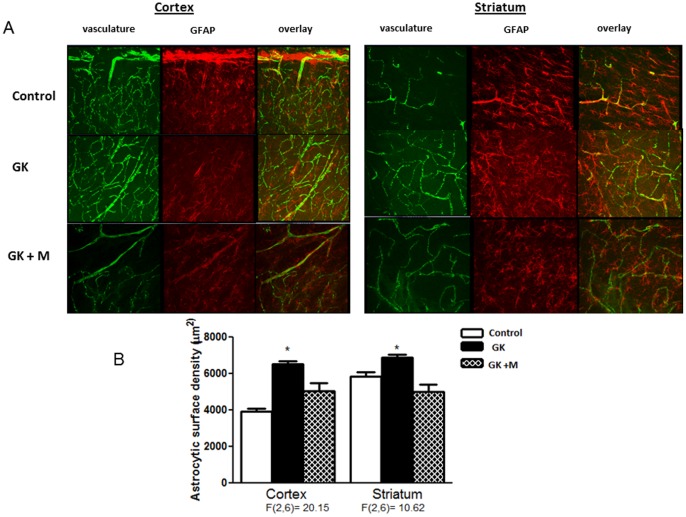
Astrocytic structural alterations are prominent in GK rats. (A) Representative images showing GFAP stained astrocytes (in red) wrapping around the vessels perfused with FITC (in green). GK rats groups have decreased number of astrocytes with more perivascular projection and smaller soma. (B) Diabetic rats show increased astrocytic surface density in both cortex and striatum compared to the control group. F values are indicated under each ANOVA analysis group. *p<0.005 vs control or treatment. Mean ± SEM, n = 3−4.

**Figure 8:**
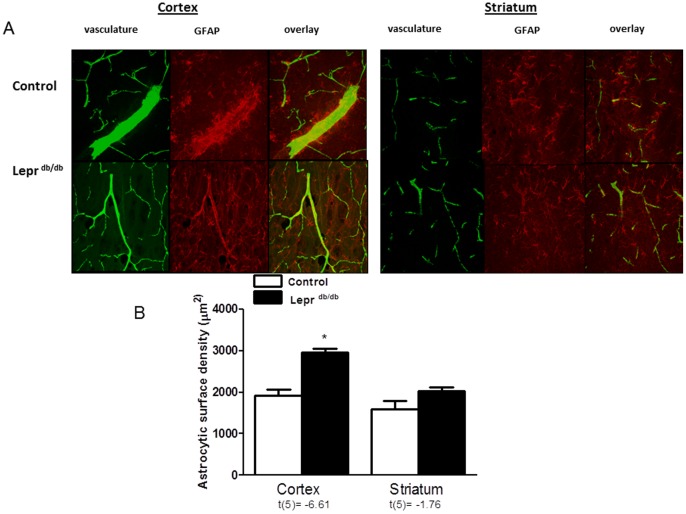
Astrocytic structural alterations are prominent in the cortex of Lepr^db/db^ mice. (A) Representative images showing GFAP stained astrocytes (in red) wrapping around the vessels perfused with FITC (in green). (B) Diabetic mice show increased astrocytic surface density in the cortex compared to the control group. t values are indicated under each analysis group. *p<0.005 vs control. Mean ± SEM, n = 3−4.

Since the PAs are surrounded by astrocytes that are critical cells in communicating the signals from neurons to the vessels for proper regulation of blood flow to provide the metabolic needs of neurons, we next assessed the presence of reactive astrocytes. There was increased surface area per astrocyte; referred to as astrocytic surface density, in both of the diabetic models (Figures 7 and 8) compared to the respective control groups. Glycemic control with metformin prevented astrocytic reactivity caused by hyperglycemia in the GK model.

## Discussion

The brain is an important target organ for complications associated with diabetes. Since changes in vascular function and structure play a major role in diabetic complications, the effect of glycemic control on cerebrovascular networking and neovascularization patterns in diabetes needs to be established. It is also important to evaluate the cerebrovascular architecture in different models of diabetes to identify commonalities and differences so that therapeutic targets and strategies can be developed for the prevention and treatment of these complications. We had previously reported that spontaneously diabetic GK rats develop dysfunctional angiogenesis shortly after the onset of diabetes. Accordingly, the objectives of this study were to determine whether 1) glycemic control prevents this erratic neovascularization, and 2) similar pathological changes occur in the Lepr^db/db^ model, which is the most commonly used model for cerebral complications of type 2 diabetes. The major findings are: 1) Glycemic control started at the onset of diabetes prevented dysfunctional neovascularization of the brain, retina and peripheral skeletal muscles in the GK rats; 2) While both microvessel and macrovessel densities in the brain are increased in GK rats, Lepr^db/db^ mice show a significant increase in the microvasculature; 3) Branch density and tortuosity of penetrating arterioles are increased in both models of diabetes; 4) Lumen diameter of penetrating arterioles are increased in GK rats; 5) Peripheral neovascularization is impaired in both models; and 6) There is increased retinal neovascularization in both models.

As recently reviewed, numerous studies reported ultrastructural changes such as basal membrane thickening, collagen deposition and endothelial degeneration in the cerebral microvasculature in multiple models of diabetes [15]. What is less clear is how diabetes impacts cerebrovascular networking and neovascularization patterns in the brain. We have recently reported that there is enhanced cerebral neovascularization that is spatially and regionally regulated in the GK model of diabetes [5]. The current study further expanded on these findings to determine the relative contribution of microvessels and macrovessels to this increased neovascularization response. In a given volume of tissue, an increase in vascular volume, which measures mainly the vascular luminal space, can be due to either remodeling of the vessel and getting larger lumen and/or due to new vessel formation. In the latter, an increase in surface area, a measure of the area the vessel wall occupies, accompanies increased volume. As such, when one looks at the relationship between volume and surface area, a linear association suggests that the increase in these two parameters go hand-in-hand and there is significant new microvessel formation. The vascularization pattern in the Lepr^db/db^ model exactly fits this model. In the GK model, however, there is extreme and disproportional increase in these parameters as a result of profuse microvasculature as well as macrovascular remodeling. Increased surface area is indicative of the large vascular surface area available for exchange of components between the tissue and the vasculature. These vessels have been already reported to have high vascular permeability [16]. Our present and published data strongly suggest that increased but dysfunctional neovascularization is specific to the brain and retina since there is decreased vascularization in the peripheral muscle. These pathological alterations in both models of diabetes may contribute to greater vascular damage and bleeding associated with stroke or neurodegenerative processes in diabetes.

Astrocytes bridge the neurovascular interaction and they have been reported to regulate cerebral microcirculatory responses and neurovascular remodeling [17], [18]. They also regulate brain metabolism and are involved in synaptic plasticity [19], [20]. Astrocytes reactivity in the hypothalamic regions and retina is increased in type 1 diabetes induced by streptozotocin [21], [22]. Astrocytes exhibit hypertrophy in the areas surrounding brain lesions and are associated with oxidative stress [23]. We report increased astrocytic reactivity in both the models of type 2 diabetes. The diabetic groups show numerous finer astrocytic processes with smaller somatic volumes compared to the control groups. The increased astrocytic reactivity together with dysfunctional cerebral vasculature may be the major contributors to increased risk of stroke, and poor vascular and functional recovery post-stroke.

Our study is the first to report an increase in the cerebral microvasculature in the Lepr^db/db^ model. A previous study showed impaired angiogenesis after stroke in this model but vascularization between control and diabetic animals in the absence of an ischemic injury was not compared in that study [24]. Previous studies have reported that the microvasculature undergoes rarefaction in the peripheral vascular beds in this model later in the disease. This study was conducted in relatively younger animals to determine the early changes in the cerebrovasculature. Whether rarefaction occurs in the brain later in the disease remains to be determined but our finding that the vascular density is already quite dramatically reduced in the skeletal muscle argues that brain vascularization is differentially regulated.

The similarities between the cerebrovascular networking patterns in these models included enhanced surface area, volume, and branch density as well as increased tortuosity. A major difference that stood out was the lumen diameter of penetrating arterioles and their first order branches, which were increased in GK rats but decreased in the Lepr^db/db^ mice. This is consistent with our findings discussed above that in the GK model; there is an increase in the % macrovasculature indicative of vascular remodeling. Diabetes and obesity leads to hypertrophic remodeling of blood vessels as observed in both older animal models and in diabetic patients [25]. The difference between models may be due to the presence of hyperlipidemia and obesity in the db/db model and or due to the differences between the glucose levels. It is also recognized that interspecies variability cannot be totally disregarded. The rationale behind our approach was to determine whether the dysfunctional neovascularization that we reported in the GK model also exist in the Lepr^db/db^ model, which is the most commonly studied model of T2D for cerebral complications of diabetes such as stroke and memory/cognitive impairment. While the use of different species with different disease severity is definitely a limitation for direct comparison of models, our results demonstrate profound and wide spread effect of diabetes on brain microvasculature.

Penetrating arterioles are considered the bottleneck of cerebral blood flow regulation as they reach deep into the brain parenchyma, they are in close association with astrocytes that provide the bidirectional communication, known as neurovascular coupling and functional hyperemia, between neurons and vessels to meet the metabolic demands of the brain [26]. In this study, the branch density and tortuosity of parenchymal arterioles are increased in both models of diabetes. Higher degree of branching and tortuous patterns has been demonstrated to be an effective determinant of blood flow changes [14], [27]. Reduced cerebral blood flow has been described in diabetic patients as well as in animal models of type 1 diabetes (T1D) [28], [29]. While we did not investigate the penetrating arteriole function in this study, we recently reported that functional hyperemia is blunted and cerebral blood flow is lower in the GK rat model [30]. Increased tortuosity and branching of penetrating arterioles may be a factor contributing to cerebral blood flow changes and need to be further confirmed in the Lepr^db/db^ model.

Glycemic control is an effective preventive strategy, as evidenced from randomized control trials such as DCCT, UKPDS, there exists a significant direct correlation between glycemia contributing to both vascular and neurological complications [31], [32]. While the impact of glycemic control on prevention of macrovascular complications is still being debated, intensive glycemic control with other agents or treatment with metformin reduced the diabetes related endpoints and microvascular complications [33], [36], [37]. Metformin in experimental animal models has shown to prevent the progression of diabetes [34], [35]. We chose to achieve euglycemia with metformin, a first line choice of drug for T2D that has been safely used with minimal side effects. We limited this treatment only to the GK rats because we wanted to determine the effect of glycemic control in a model in which we know there is established pathological neovascularization. Early glycemic control with metformin abrogated abnormal responses like cerebral neovascularization, tortuosity and branching mediated by diabetes. Metformin was also effective in preventing peripheral vascular regression and retinal hypervascularization emphasizing beneficial role of glycemic control. Taken together, this study highlights the possible beneficial role of the glycemic control in preventing cerebral vascular disease in diabetes.

In conclusion, while there are some differences in the vascularization patterns between the GK and the Lepr^db/db^ models, the brain vasculature is an early target in diabetes. Increased yet dysfunctional cerebral vascularization is similar to what is observed in diabetic retinopathy and suggests that vascular maturation and pruning processes may be impaired in diabetes. Better understanding of the dynamic changes that occur in the cerebrovasculature with the progression of diabetes and underlying mechanisms are likely to yield novel approaches to prevent and/or treat cerebral complications of diabetes.

## Supporting Information

Figure S1 Schematics showing tissue procession performed using Fiji and Volocity software explained in the methodology.
(TIFF)
