# Supplementary figures and images for: Cerebral Neovascularization and Remodeling Patterns in Two Different Models of Type 2 Diabetes
Source: PLoS One. 2013 Feb 18;8(2):e56264. doi: 10.1371/journal.pone.0056264 (PMC3575336; doi:10.1371/journal.pone.0056264)

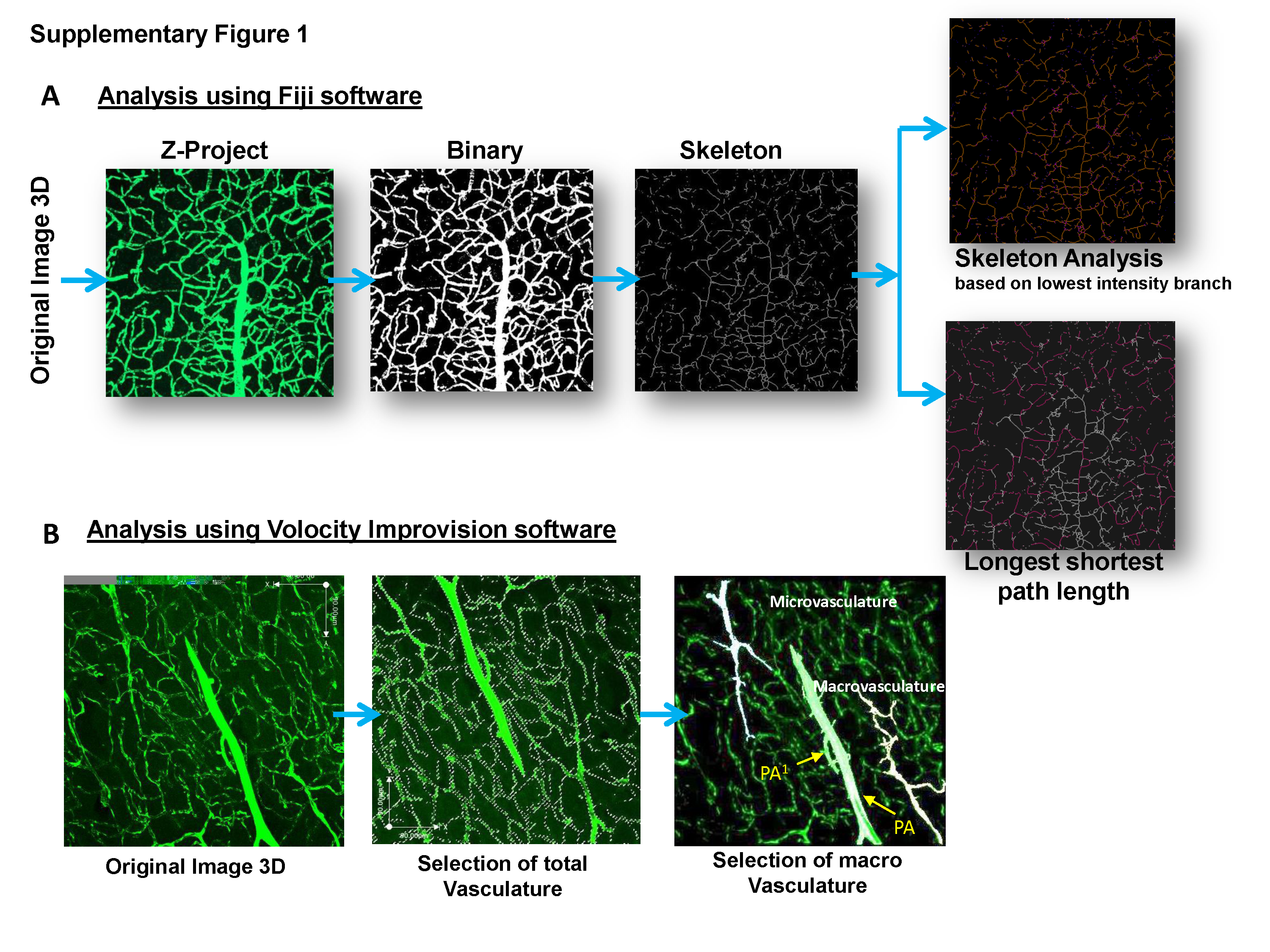

Supplement: Figure S1 — Schematics showing tissue procession performed using Fiji and Volocity software explained in the methodology. (TIFF) [file pone.0056264.s001.tiff]
